# Sensing Mercury for Biomedical and Environmental Monitoring

**DOI:** 10.3390/s90705446

**Published:** 2009-07-09

**Authors:** Paul D. Selid, Hanying Xu, E. Michael Collins, Marla Striped Face-Collins, Julia Xiaojun Zhao

**Affiliations:** 1 Department of Chemistry, University of North Dakota, Grand Forks, ND 58202, USA; 2 Environmental Analytical Research Laboratory, University of North Dakota, Grand Forks, ND 58202, USA; 3 Tribal Environmental Science Department, United Tribes Technical College, Bismarck, ND 58504, USA

**Keywords:** determination of mercury, sensors, nanomaterials, mercury pollution, fluorescence

## Abstract

Mercury is a very toxic element that is widely spread in the atmosphere, lithosphere, and surface water. Concentrated mercury poses serious problems to human health, as bioaccumulation of mercury within the brain and kidneys ultimately leads to neurological diseases. To control mercury pollution and reduce mercury damage to human health, sensitive determination of mercury is important. This article summarizes some current sensors for the determination of both abiotic and biotic mercury. A wide array of sensors for monitoring mercury is described, including biosensors and chemical sensors, while piezoelectric and microcantilever sensors are also described. Additionally, newly developed nanomaterials offer great potential for fabricating novel mercury sensors. Some of the functional fluorescent nanosensors for the determination of mercury are covered. Afterwards, the *in vivo* determination of mercury and the characterization of different forms of mercury are discussed. Finally, the future direction for mercury detection is outlined, suggesting that nanomaterials may provide revolutionary tools in biomedical and environmental monitoring of mercury.

## 1. Introduction

Mercury generally adopts one of three common forms: elemental mercury (Hg^0^), ionic mercury (Hg^2+^), and organic mercury complexes. Organic mercury complexes mainly include methylmercury, dimethylmercury and phenylmercury, with methylmercury (CH_3_Hg^+^) being the most toxic of all forms to living systems. Depending on environmental conditions, mercury can transform among the different forms, so the existence of any form of mercury is potentially harmful to human health. Unfortunately, research results indicate that mercury emissions have increased relative to natural sources since the beginning of the industrial era [1–3]. Average mercury levels in the atmosphere are 3–6 fold higher than the pre-industrial estimates. The increase in environmental mercury is best attributed to anthropogenic sources [3]. Industrial processes tend to release geologically bound mercury from mercury reservoirs into the atmosphere as elemental mercury. Once in the atmosphere, elemental mercury oxidizes into ionic mercury and deposits in the environment, possibly causing elevated mercury levels.

Concentrated mercury levels pose serious health problems. Bioaccumulation creates harmful levels of mercury towards the top of the food chain. Consumption of species near the top of the chain can result in high levels of mercury within the brain and kidneys, ultimately leading to neurological diseases. Therefore, monitoring mercury is important for environment and human health.

A wide variety of mercury determination techniques has been developed. The majority of these techniques are based on analytical instrumentation methods. Two very popular methods are cold-vapor atomic absorption spectrometry and atomic fluorescence spectrometry. These methods can determine mercury with very high sensitivities. In addition to analytical instruments, various mercury sensors provide a convenient means to determine both abiotic and biotic mercury [4–7]. In particular, newly developed photoactive nanomaterials present an exciting and truly revolutionary approach to mercury detection [8–13]. This article initially discusses the current problems that mercury poses on the environment and human health. Then, it focuses on present mercury sensors with an emphasis on biosensors and chemical sensors. Finally, the article briefly reviews the potential techniques for *in vivo* mercury detection and the ability to identify different forms of mercury.

## 2. Mercury Pollution and Hazard to Human Health

Natural mercury emissions have led to the distribution of mercury throughout the environment. Volcanoes, fires, rivers, and biological processes can all serve as the primary vehicles for this distribution [3]. Off-gassing of mercury from the lithosphere and hydrosphere to the atmosphere results in the deposition of mercury in aquatic and terrestrial environments. In addition to the natural mercury emissions, human activities and the advent of industry have created new pathways for mercury emissions.

Human-related mercury emissions such as mining of coal and silver [14], burning of fossil fuels, and industrial processes have increased with respect to natural emissions. The emitted mercury is released to various sites in the environment. Approximately 80% of anthropogenic mercury emissions release elemental mercury (Hg^0^) into the air through industrial processes. Meanwhile, almost 15% of this mercury is released into the terrestrial environment. The final 5% of anthropogenic mercury emissions is transported from industrial wastewater to the aquatic environment [6]. A total estimate of 4,700 tons of mercury is released from human-related activities each year to deposit in the environment [4]. Deposited mercury can then re-emit into the atmosphere by biological and geological means.

Mercury concentrations in ambient air in the USA range from 5 × 10^−14^ M to 1 × 10^−13^ M. Increased levels of mercury as high as 5 × 10^−11^ M to 7 × 10^−11^ M are found in industrialized areas. In contrast, in some less-polluted areas of the world such as Sweden, the elemental mercury range is 1 × 10^−14^ M to 3 × 10^−14^ M [7], exhibiting much lower levels of mercury. Thus, to control mercury pollution, the reduction of human-related mercury emissions is critical.

The transformations and cycles of mercury within the environment have been thoroughly studied [15,16]. Mercury can undergo complex transformations within the human body. Elemental mercury is absorbed through the lungs while ionic mercury is absorbed through the intestines. The elemental mercury species are commonly oxidized to divalent ionic mercury and target the brain and kidneys. In general, elemental mercury is more easily transported across the blood-brain barrier than ionic mercury.

The primary means of mercury exposure is through the consumption of aquatic organisms (i.e., fish). Mercury concentrations within freshwater fish are in the range of 1.5 × 10^−6^ to 2.0 × 10^−6^ M whereas the concentrations of mercury within oceanic fish are in the range of 3.0 × 10^−6^ M to 4.0 × 10^−6^ M [4]. Additional exposure may result from the consumption of wild mammals [4]. Meanwhile, dental amalgam fillings also pose a potential source of exposure to mercury. When exposed to the mouth environment (chewing and grinding), very small amounts of mercury are released into the human body, ranging from 1 × 10^−8^ M to 8 × 10^−8^ M per day [6]. Mercury can also enter the human body through breathing mercury vapors. Once in the body, mercury first enters the blood stream via lungs and accumulates in and around the blood-brain barrier where severe neurological diseases can pursue from metal-induced toxicities [6,17].

Methylmercury is a highly neurotoxic species. Most of the methylmercury within the blood is bound to proteins and sulfhydryl-containing groups. The small complexes can mimic the behavior of endogenous substrates; therefore, they can gain access to the brain via the transport system across the blood-brain barrier [17]. Symptoms of such poisoning include personality changes, tremors, loss of sensation, and muscle coordination difficulties [3,6].

## 3. Sensitive Determination of Mercury

A wide variety of instrumental methods has been developed for the determination of environmental mercury. Sophisticated analytical techniques include atomic absorption spectrometry, atomic emission spectrometry, and inductively coupled plasma/mass spectrometry. These instrumental methods provide high sensitivities for monitoring trace amounts of mercury in the environment. Among them, cold-vapor atomic absorption spectrometry is frequently used to accurately measure mercury due to its simplicity and good reproducibility. However, the original forms of mercury in the sample are destroyed in the process of such techniques. Thus, the detected amounts of mercury are total forms of mercury including Hg^0^, Hg^2+^, CH_3_Hg^+^ and other organic mercury complexes. A challenge exists to differentiate mercury forms in various samples using the instrumentally-based methods.

Several sensors have proven to be effective tools for monitoring different forms of mercury including biosensors, chemical sensors, nanosensors, microcantilever sensors and piezoelectric sensors. These sensors usually detect abiotic mercury. Since the majority of mercury in the environment is abiotic mercury, the sensors are appropriate tools to monitor environmental mercury as described below. However, the *in vivo* monitoring biotic mercury is a great challenge. The initial effort on this regard is briefly covered in the last paragraph of this section.

### 3.1. Biosensors

Mercury can be selectively determined using DNA sequences. Several mercury forms, including inorganic and organic types, can bind to DNA and result in conformational changes in the DNA structure [18–22]. The binding affinity of mercury complex with DNA is in the order MeHg^+^∼PhHg^+^>EtHg^+^>Hg^2+^ [18]. The interactions of mercury with DNA can be probed by capillary electrophoresis (CE), and analyzed with electrothermal atomic absorption spectrometric detection (CE-ETAAS), infrared spectrometry and circular dichroism.

Hg^2+^ tends to bind to thymine-thymine (T-T) base pairs in DNA structures [19–22], and then induces a DNA conformational change. The change is dependent upon the sequence of base pairs but tends to form a hairpin structure [23]. A common theme in this area of experimentation is to attach a fluorophore to one end of the DNA sequence with a quencher on the opposite end. Upon formation of the hairpin structure, the fluorescence of the fluorophore is quenched due to fluorescence resonance energy transfer (FRET) [21,22].

When mercury binds to porphyrins forming a porphyrin-mercury complex, the resultant difference in DNA conformational changes is much greater, suggesting that the porphyrin-mercury species are more harmful than the free metal ion counterparts [23].

Interference analysis with other common metal ions suggests that the T-T interactions are specific to mercury [19,21,22]. Usually, Cd^2+^ and Pb^2+^ can induce different conformational changes in the DNA structure than interactions with Hg^2+^. Thus, the biosensors based on DNA conformational changes have high selectivity.

In addition to the sensors dependent upon DNA sequences, recombinant whole cell bacterial sensors for the detection of organic and inorganic mercury have also been studied extensively. Genetically modified *E. coli* strains containing a lacZ reporter gene linked to the mercury-responsive zntA were used to create an Hg^2+^ selective biosensor [24]. Hg^2+^ induced a fluorescence response upon interaction with the zntA promoter. Similar studies were performed using the copA promoter that specifically responds to other heavy metals [25].

**Figure 1.**
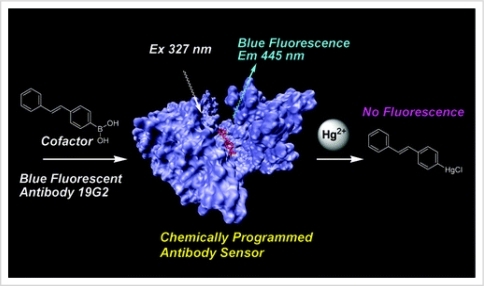
A EP2-19G2-cofactor biosensor for mercury. Reprinted with permission from [29]. Copyright American Chemical Society (2005).

The idea of recombinant whole bacterial cell sensors has been expanded to determine a wider range of mercury species. The mercury-inducible mer operon is induced by organic and inorganic mercury. The mer operon controls the luciferase gene. The response of the luciferase gene was observed and deemed capable of measuring Hg^2+^, MeHg^+^, PhHg^+^, and Me_2_Hg [26]. The recombinant bacterial sensors with the mer operon have proven to function properly in measuring real-life samples in the presence of other metal interferences [27,28]. A schematic diagram of determination of Hg^2+^ based on antibody-antigen reactions is shown Figure 1. When bound to a cofactor, a chemically programmed antibody exhibited fluorescence. Upon the addition of Hg^2+^, the fluorescence was quenched because the Hg^2+^ bound to the cofactor and formed a non-florescent compound. This sensor was specific for Hg^2+^ in the presence of other interfering cations [29].

### 3.2. Chemical Sensors

Chemical sensors are also used to determine mercury. Due to the high sensitivity that can be achieved with the technique, the most common chemical sensors are based on fluorescence signals. Chemical sensors offer the unique advantages of a long lifetime and low costs.

A new chemical sensor for the determination of ionic mercury is based on the fluorescence quenching of a sol-gel membrane [30]. The membrane worked according to an ion-exchange mechanism in which Hg^2+^ bound to a porphyrin immobilized on a sol-gel membrane. The binding of Hg^2+^ quenched the fluorescence signal of the porphyrin; therefore, the change in fluorescence intensity was proportional to the mercury concentration.

The development of target-induced fluorescence sensors has attracted considerable attention in heavy-metal detection due to excellent selectivity and sensitivity. The target-induced sensors are generally based on Hg^2+^ desulfurization reactions, such as cyclizations, hydrolysis, and elimination reactions. The Hg^2+^-promoted desulfurization reaction of a thiocarbazone derivative yields a cyclic product, upon which a fluorescence enhancement is generated [31]. Interference analysis for other cations reveals a specific interaction between the thiocarbazone derivative and the Hg^2+^.

**Figure 2.**
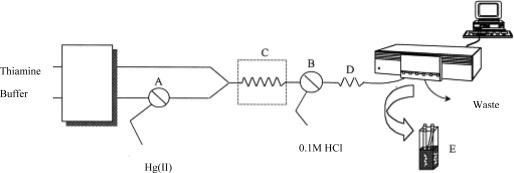
Schematic diagram of a flow system in a mercury chemical sensor. A and B, valves; C, thermostatted water bath; D, cooling coil; E, fluorescence flow cell. Reprinted with permission from [32]. Copyright Elsevier (1999).

A chemical sensor combined with a flow injection system can continually measure mercury in the environment. For example, a sensor based on a non-ion exchanging solid support with thiamine was developed to selectively and sensitively determine ionic mercury (Figure 2) [32]. The principle of the method was the oxidation of thiamine to fluorescent thiochrome. The mercury induced fluorescence signal was proportional to the mercury concentration.

**Figure 3.**
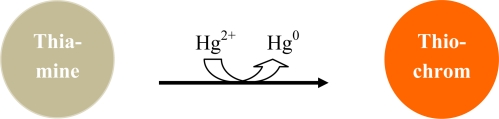
Ionic mercury reacts with thiamine to generate a “turn-on” fluorescence signal when thiochrome is formed.

The design of fluorescence markers upon the addition of Hg^2+^ is of considerable interest. Few markers are available for detection of mercury in a competitive environment. Sensitivity, selectivity, and solubility must all be addressed by the sensor. Achieving all criteria is a challenge, but fabrication of such sensors has been reported. A fluorescence molecular sensor for Hg^2+^ is based on a phosphane sulfide derivative. The detection limit of 3.8 × 10^−9^ M was achieved while retaining a high selectivity over competing cations in an aqueous medium [33]. Selective chemodosimeters for mercury have been developed. Mercury-triggered intra-molecular cyclizations of thioureas result in the formation of highly fluorescence molecules [27]. Other fluorescence markers with attached receptors specific to ionic mercury exhibit an enhanced fluorescence upon the addition of Hg^2+^ [35–37]. Thiamine (Vitamin B_1_) acts as a “turn-on” fluorescent marker specific to ionic mercury. As mercury interacts with thiamine, thiamine is oxidized to thiochrome, and mercury is reduced to elemental mercury (Figure 3). Overall fluorescence sensors offer a selective and sensitive approach for the determination and monitoring of mercury in an aqueous medium [36].

### 3.3. Conductometric Sensors, Microcantilever Sensors

Mercury has a specific affinity with gold. The conductivity of gold can be utilized to fabricate of mercury sensors. These conductometric sensors have shown excellent sensitivity for sensing elemental mercury vapor [38]. The vapor mercury is adsorbed on the surface of thin gold film and produces a resistance change in the film. Most recently, a sintered thick PdCl_2_ film_,_ in addition to gold film, has been employed for the detection of vapor mercury with better regeneration capability [39]. A comparison of several sensing materials for the monitoring of elemental mercury vapor was summarized by Shevade and co-workers [40].

**Figure 4.**
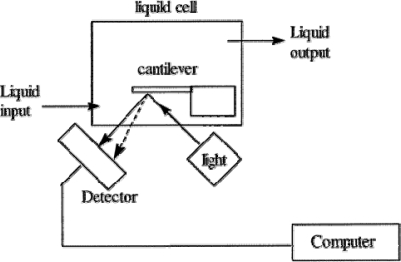
Schematic diagram of a microcantilever sensor system. Reprinted with permission from [42]. Copyright American Chemical Society (2002).

Microcantilever sensor is a relative new technique for the determination of mercury. Several effective microcantilever sensors have been developed for the determination of ionic and elemental mercury [41–43]. A thin film of gold is coated onto the microcantilever that undergoes a slight bend when the mercury deposits on the gold surface. Based on the degree of bending, the sensor can determine the amount of mercury present in the solution (Figure 4). An *in situ* detection of mercury using the microcantilever was demonstrated with a high sensitivity and selectivity. The results showed that the gold-coated cantilever responds to ionic mercury concentrations as low as 1 × 10^−11^ M of Hg^2+^ [42].

### 3.4. Surface Acoustic Wave (SAW)-Based Sensor and Piezoelectric Detection

Surface acoustic wave (SAW) based sensors offer great potential for reproducible detection of gaseous elemental mercury [44,45]. The SAW sensor can be made of gold film [44] or semi-conductive materials [45]. The principle of the SAW sensor is well introduced in the literature [46]. In short, the surface acoustic wave energy produced in the sensor system is confined to the surface of gold. The depth of the wave penetrated to the SAW substrate is only one wavelength. This characteristic makes the SAW very sensitive to any changes on the gold surface. As the mercury vapor flow through the surface of the sensor, the elemental mercury reacts with the surface materials and results in the change of surface oscillation frequency. Based on the frequency of the oscillations, the concentration of mercury is determined.

Similar to SAW sensors, piezoelectric detection based sensors recognize elemental mercury [47]. The principle of piezoelectric sensor is that the vibration frequency of a piezoelectric crystal decreases when the mercury is absorbed on the sensor surface. This decrease is proportional to the amount of mercury absorbed [47]. Usually, the targeted mercury species in aqueous solution are first reduced and deposited on a gold-plated piezoelectric crystal. Then, the system allows for a simple and rapid yes/no binary response to mercury. Piezoelectric detection is beneficial for *in situ* measurements using portable equipment. Recently, an automated mercury microgravimetric screening system based on the piezoelectric detection was reported [48]. The detection limit was as low as 1 × 10^−9^ M of Hg^2+^.

### 3.5. Nanosensors

**Figure 5.**
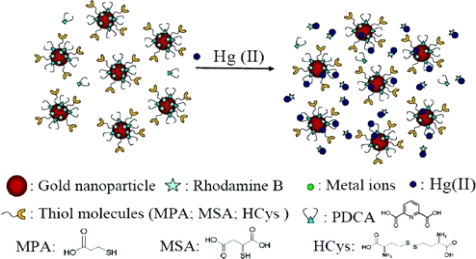
Rhodamine B-AuNP mercury sensor modified by thiol ligands and PDCA. Reprinted with permission from [8]. Copyright American Chemical Society (2006).

The advent of nanomaterials offers great potential for a more selective, sensitive, and rapid determination of mercury. Several nanomaterials have been used for monitoring mercury. Gold nanoparticles and gold nanorods are the primary means of incorporating nanomaterials into mercury detection [8–10]. For example, a gold nanoparticle (AuNP) was functionalized with a fluorescent molecule, rhodamine B (RB), for the determination of ionic mercury in aqueous solutions (Figure 5). The sensor was based on a “turn on” fluorescence signal upon the presence of ionic mercury. Before the mercury appeared in the solution, the fluorescence signal of rhodamine B was quenched by the gold nanoparticles when the distance between the gold and the fluorescent molecules is less than 10 nm. The ionic mercury released the rhodamine B from the gold nanoparticle surface and thus restored the fluorescence signal of rhodamine B. The amount of the released rhodamine B was proportional to the concentration of mercury. The selectivity of the rhodamine B-AuNP sensor for mercury was improved by modifying rhodamine B-AuNP surfaces with thiol ligands (MPA, MSA, and HCys) and adding a chelating ligand (PDCA) to the sample solutions [8].

A gold nanoparticle-based mercury sensor, functionalized with rhodamine 6G, achieved a detection limit as low as 6.0 × 10^−11^ M of Hg^2+^ [9]. The gold nanoparticles were soluble in aqueous solutions. The surface modification of the gold nanoparticle improved selectivity of the sensors. The nanoparticle approach also offered a rapid determination. The mercury concentration was obtained within 10 min [8].

A different type of gold nanomaterial, gold nanorods, was used to detect mercury in tap water. Using an amalgamation between mercury and gold, the high selectivity and extraordinary simplicity for determination mercury was achieved, making gold nanorods a great candidate for mercury detection [10]. Additionally, nanostructure cage materials [11], gold nanowires [12], and self-assembled nanoparticle probes [13] offer further insights into mercury detection using nanomaterials.

### 3.6. In vivo Monitoring Mercury

Non-invasive *in vivo* monitoring of mercury is of great importance in biological and medical studies. To date, some *in vivo* determinations of mercury have been developed [49–53]. The measurement based on X-ray fluorescence [49–51] is a remarkable example. The previous detection limit of X-ray fluorescence method was 19 ppm for a kidney [49], which is too high to be used for clinical purposes. The most recent work reported by Grinyer’s group [50] greatly improved the sensitivity of X-ray fluorescence measurement. A detection limit of 5.0 ppm mercury at a 1 cm phantom depth was achieved in a kidney sample.

Some fluorescence chemical sensors also have the potential to detect mercury within living cells and vertebrate organisms. Tae and Shin’s research group described an irreversible rhodamine-based chemical sensor for *in vivo* monitoring mercury ions in living cells. Using this system, they monitored the accumulation of mercury ions in zebrafish tissues and organs. The rhodamine-based sensor undergoes a cyclization reaction to generate a strongly fluorescent molecule that was sensitive to mercury in the ranges of 0.1–8.0 ppm. The results suggested that a 1:1 stoichiometric relationship existed between mercury and the fluorescent molecules for quantitative detection of mercury [53].

The underlying problem with determining mercury *in vivo* is the ability to distinguish different forms of mercury. The techniques discussed above are able to measure total mercury levels without characterizing the forms of mercury within samples. A more complete development of a sensitive, safe, and non-invasive method to monitor mercury is still needed.

### 3.7. Characterization of Different Forms of Mercury

Identification of different forms of mercury is important for mercury analysis. In addition to sensors, great efforts also are being made to develop instrumental methods for the simultaneous identification and determination of various mercury forms. Currently, two steps are needed in the instrumental methods. The first step is the separation that is usually based on gas chromatography (GC), high-performance liquid chromatography (HPLC), or capillary electrophoresis (CE) [54,55]. The second step is the determination using elemental detection methods, such as atomic absorption spectrometry, atomic emission spectrometry, atomic fluorescence spectrometry [55,56], inductively-coupled plasma/mass spectrometry (ICP-MS) [57,58], and cold-vapor atomic absorption spectrometry. One danger in this effort might be in the process of extraction of mercury from the sample matrix [59]. The extraction tends to transform inorganic mercury to methylmercury [54,60]. Overall, the development of instrumental methods is still in process.

## 4. Future Directions

The application of nanomaterials for the sensitive determination of mercury is in the initial stage. Based on current results, nanoparticles may perform *in vivo* determination with high sensitivity and selectivity. Meanwhile, characterizing different forms of mercury is greatly needed. The ability to confidently, selectively, and sensitively detect mercury in all its various forms allows researchers to better understand the transformations and cycles of mercury within the environment.

In addition to determination, bioremediation of mercury is important to reduce the mercury hazard to human health. Bioremediation technology uses microorganisms to eliminate or control the amount of contaminants in the aquatic and terrestrial environments. Several methods for bioremediation of mercury are proposed, which are focused on the reduction of ionic mercury (mainly concentrated in the aquatic system) to elemental mercury. The reduction is carried out through the cytoplasmic enzyme mercuric reductase (encoded from the gene mer A). This method offers an environmentally friendly approach to remove mercury from the aquatic environment or transform mercury to more insoluble, less-toxic species [61].

## 5. Conclusions

A number of sensors for sensitive and selective monitoring of mercury have been developed. A comparison of several typical mercury sensors is listed in the Table 1.

Biosensors are attractive sensing methods for effective detection of mercury. For instance, probing mercury species with DNA sensors offers a unique approach for the detection and characterization of different forms of mercury. Chemical sensors are regarded as robust sensors with optimal sensitivity. Non-invasive *in vivo* detection of mercury is becoming more important as environmental mercury levels continue to rise. Major advancements in X-ray fluorescence allow for *in vivo* monitoring mercury but greater developments are still needed. The emerging field of nanotechnology offers the potential to develop more sensitive and selective methods to detect mercury. Further developments in nanotechnology will improve mercury determination methods and grant a better understanding of mercury transformations and cycles in environmental and biological processes.

## Figures and Tables

**Table 1.** A comparison of different types of mercury sensors.

**Type of sensor**	**Sensing principle**	**Fabrication of the sensor**	**Forms of mercury detected**	**Detection limit**	**Ref.**
Biosensor	Mercury interaction with bacterial cell	Moderately difficult	Inorganic Hg Organic Hg	∼10^−7^M	[24]
	Mercury interaction with antibody	Moderately difficult	Hg^2+^	∼10^−6^ M	[29]
Chemical sensor	Fluorescence quenching	Moderately difficult	Hg^2+^	∼10^−6^M	[30]
	Fluorescence enhancing	Moderately difficult	Hg^2+^	∼10^−9^M	[31–33]
Conductometric sensor	Conductivity/resistance	Easy	Hg vapor	∼10^−8^ M	[38–40]
Microcantilever sensor	Physical property changes	Easy	Hg^2+^, Hg^0^	∼10^−11^ M	[41–43]
SAW sensor	Oscillation frequency	Easy	Hg vapor	∼10^−8^ M	[44]
Piezoelectric sensor	Frequency of vibration	Easy	Hg^0^	∼10^−9^M	[47–48]
Nanosensor	Interaction with nanoparticles	Moderately difficult	Hg^2+^	10^−11^∼10^−15^ M	[9–10]
